# Factors Associated With Marital Satisfaction in Infertile Couple: A Comprehensive Literature Review

**DOI:** 10.5539/gjhs.v8n5p96

**Published:** 2015-08-31

**Authors:** Keshvar Samadaee-Gelehkolaee, Barry W McCarthy, Alireza Khalilian, Zeinab Hamzehgardeshi, Sepideh Peyvandi, Forouza Elyasi, Maryam Shahidi

**Affiliations:** 1 Department of Reproductive Health and Midwifery, Nasibeh Nursing and Midwifery Faculty, Mazandaran University of Medical Sciences, Sari, Iran; 2 Student Research Committee, Mazandaran University of Medical Sciences, Sari, Iran; 3 Department of Psychology, American University, Washington, DC, USA; 4 Department of Biostatistics, Sari Medical School, Mazandaran University of Medical Sciences, Sari, Iran; 5 Traditional and Complementary Medicine Research Centre, Mazandaran University of Medical Sciences, Sari, Iran; 6 OB/GYN Department, Imam Khomeini Hospital, Mazandaran University of Medical Sciences, Sari, Iran; 7 Department of Psychiatry, Psychiatry and Behavioral Sciences Research Center, School of Medicine, Mazandaran University of Medical Sciences, Sari, Iran; 8 Department of Medical Physics, Mazandaran Medical University, Mazandaran, Iran; 9 Hazrat-e Maryam Fertility Center, Sari, Iran

**Keywords:** marital satisfaction, sexual satisfaction, infertile couples, factors associated

## Abstract

**Background:**

Many factors impact on marital satisfaction. Related factors include demographic factors, assisted reproductive techniques, psychological health, quality of life, psychological, socioeconomic and family support, and sexual function.

**Methods:**

This study is a literature review of research studies conducted on factors associated with marital satisfaction in infertile couples. The current literature review search was undertaken using multiple databases selected from articles pertinent to the study. The selection of subjects was undertaken from1990 through 2015. The methodological quality was analyzed based on a checklist adopted from a systematic review. Quality assessment of full text studies was finally carried out by two reviewers.

**Results:**

The initial search yielded a list of 445 papers, and then reviewers studied titles and abstracts. Thereafter, 69 papers were incorporated, and researchers reviewed summaries of all of the searched articles. Finally, the researchers utilized the data gained from 64 full articles so as to compile this review paper. Reviewing the studies conducted on marital satisfaction, they classified related findings into 6 categories: demographic factors, using fertility assisting methods, psychological health, life quality, economic, social, and family support, and sexual function.

**Conclusion:**

The results of this review article depicted that various factors play role in creating marriage life satisfaction in an infertile couple, so that paying attention to them can play an important role in continuing their treatment. Thus, to identify such factors is considered essential in their treatment protocol highly based on culture. Of the drawbacks of this research is that it has tried at best to employ the studies belonging to diverse countries with different cultures. Also, the number of the papers was considerably limited.

## 1. Introduction

Infertility can have major effects on a couple’s life, including marital satisfaction, and has a remarkable role in family life and welfare (Moura-Ramos, Gameiro, Canavarro, & Soares, 2012). Marital satisfaction refers to how the sexual partners’ expectations from each other are met (Mirghafourvand, Charandabi, Jafarabadi, Tavananezhad, & Karkhane, 2013), the reduction of which can render adverse effects on the couple’s body and mind (Kazemi, Aghamohammadian Sherbaf, Modarres Gharavi, & Mahram, 2011). Various factors are involved in marital satisfaction, including the demographic ones (Shakerian, 2010), hardiness (Shivarani, Fallah, & Allahyarri, 2011), intimacy (Greeff & Malherbe, 2001) sexual function (Rahmani, Khoei, & Gholi, 2009; Shakerian, Nazari, Masoomi, Ebrahimi, & Danai, 2014), marital conflict (Amrelahi, Roshan Chesly, Shairi, & Nik Azin, 2013), and stress (Randall & Bodenmann, 2009). However, the couple World Health Organization (WHO) doesn’t have a child after one year of unprotected intercourse may be more affected in terms of their marriage contentment, since in many couples, not being able to have their biologic child is considered as a personal tragedy (Marci et al., 2012; Reis, Xavier, Coelho, & Montenegro, 2013). This issue deserves consideration from two aspects: first, the fact that 10-15% of the world’s population are challenging with infertility and seeking treatment assisted fertility methods in order to realize their dreams so that in the U.S, out of every 100 born infants, one is born through reproduction assisting methods (Cwikel, Gidron, & Sheiner, 2004; Galhardo, Cunha, & Pinto-Gouveia, 2013). On the other hand, infertility, through impacting on marital satisfaction or through aggravating marital relationships can directly or indirectly bring about failure in fertility (Faria, Grieco, & Barros, 2012). Accordingly, knowing the factors associated with marital satisfaction in the infertile couples, it is possible to help them keep on their treatment and increase their success chance through planning for effective interventions (Hughes & da Silva, 2011; C. A. Smith et al., 2012). Thus, and the present study aims to analyze the factors related to marital satisfaction in infertile couples.

## 2. Method

The present literature review adhered to the following four steps: 1. Identifying the research question; 2. Searching methods to identify relevant studies; 3. selecting the study; 4. charting the data ‘collating, summarizing, and reporting the results (Hamzehgardeshi, Shahhosseini, & SamadaeeGelehkolaee, 2015).

The protocol of the study was approved by the Ethics Committee of Mazandaran University of Medical Sciences, Ethics No. 16/2/1394-1409.

### 2.1 Identifying the Research Question

What are the related factors associated with marital satisfaction in infertile couples?

### 2.2 Searching Methods to Identify Relevant Studies

The researchers utilized Google Scholar general search engine, and later more specifically Science Direct, ProQuest, SID, Magiran, Irandoc, Pubmed, Scopus, Cochrane library, Psych info, Cumulative Index to Nursing, and Allied Health Literature (CINAHL). Search strategy was performed using the following keywords as well as their Farsi equivalents. Medical Subject Headings terminology (MeSH) was used where possible (in Pubmed), and keywords used in those databases were not used in Medical Subject Headings terminology. The terms used were: “Infertile couples or infertile spouse”, “marital satisfaction”, “marital relationship”, “marital status”, “related factors”, “risk factors”, “sexual satisfaction”, “sexual relationship”, and “sexual status”. Selection of the subjects of the articles relevant to the study was carried out from 1999 through 2015.

### 2.3 Selecting the Study

**The initial search yielded** a list of 445 papers, and then reviewers studied titles and abstracts. Thereafter, 69 papers were incorporated, and researchers reviewed summaries of all of the searched articles. Thereafter, quality assessment of full text studies was performed by two independent reviewers. Researchers reviewed summaries of all articles sought. Finally data extracted from 64 full articles were used to compile this review paper. The inclusion of all titles and abstracts, in English and Persian languages, was assessed by researchers.

#### 2.3.1 Inclusion Criterion

Peer-reviewed articles published between 1990 and 2015 were included, which described the relationship between marital satisfaction and related factors contributing to infertility and measured marital relationship with a validated instrument and compared two or more fertile and infertile groups with one another.

#### 2.3.2 Exclusion Criterion

Papers describing the relationship between marital satisfaction and related factors in fertile couples were excluded.

#### 2.3.3 Quality Assessment

The studies’ quality was analyzed based on a checklist in Table 1 adopted from a systematic review. This checklist includes 16 items, and if the studies include each of the items, they are assigned score 1, if that item does not exist in the study or insufficient data is offered, it is given score 0. And finally, the scores sum 16 has been calculated where each study with 75% criteria (12-16 scores) possesses high quality, the ones with 50-75 % criteria(between 8 and 12 points) have average quality, and the studies having less than 50% criteria (below 8 scores) have poor quality.

**Table 1:** Check List of criteria for assessing the quality of studies on marital relationship in the infertile

A. a psychometrical questionnaire is applied
B. a chief objective of the study is to investigate the marital relationship
C. standardized or valid self-report measurements are utilized to assess the marital relationship in the infertile and/or their spouse/partners.
**Study participants**
D. a description consists of at least two socio-demographic variables (e.g., age, sex, economical status educational status, etc).
E. a description presents at least two clinical variables (e.g., type of infertility, duration of infertility, treatment method(s), etc).
F. inclusion and/or exclusion criteria are provided
G. the study describes predictors or contributing factors using correlation analyses, multivariate analyses, or structural equation models)
H. rates of participation for the infertile groups and/or their spouses/partners are described (defined as the percentage of eligible patients giving their informed consent) and these rates exceed 70%
I. information is provided about the ratio between non-responders versus responders.
**Study design**
J. the study size is consisting of at least 50 patients
K. the collection of data are prospectively gathered and cross-section.
L. the design is longitudinal (more than 1 year)
M. the process of data collection is described (e.g., interview or self-report, etc.)
N. the follow-up period is at least 6 months
O. the loss to follow-up is described and is less than < 20%.
**Results**
P. the results are compared between two groups or more (e.g., healthy population, groups with different treatment stages, different types of infertility, or treatment types) and/or results are compared with at least two points in time (e.g., pre- versus post- treatment)

Ref. (Tao, Coates, & Maycock, 2011).

#### 2.3.4 Data Collection and Analysis

2.3.4.1 Selecting the Studies

For the inclusion of all titles and abstracts identified during the literature searches was assessed by one author reviewing the search results and identifying reports for inclusion or exclusion. The reports identified for appropriate categorization were also examined by another author.

2.3.4.2 Extracting and Managing Data

The data based on the quality of articles (Table 1) were extracted and the information was entered into tables. Another author conducted a second data extraction and verified correct data entry.

The criteria checklist had its basis on an established criterion for systematic review of the reports in the literature (Tao, Coates, & Maycock, 2011).

### 2.4 Charting, Collating, and Summarizing the Data

Data extracted were summarized in Tables 2 and 3.

**Table 2:** Marriage Satisfaction Related Factors

Marriage Satisfaction Related Factors	Related papers No.	Including	Action Mechanism	Recommendations
Demographic	13	Age, gender, education, marriage duration, infertility duration, previous child, family type (nuclear, extended), income	Via support & stress determination, couple’s relationships & marriage satisfaction change.	Depending on the infertility cause (male-female) & duration (more than 2 yrs), the couple needs various kinds of consultation, for example, male infertility requires sexual consultation.
Using fertility assisting methods	11	Taking medicine, embryo donation, gamete donation, IUI, IVF, ICSI	Due to drug side-effects induced stresses & not psychologically & morally adapting with embryo & gamete donation acceptance, especially when there is conflict between the couple.	Education about the medicinal short-& long-term effects, education about the invasive methods steps and their due risks, psychological & religious consultation, in case of failure in treatment, women’s follow-up for 6 months in terms of depression & anxiety.
Psychological health	12	The probability of getting affected by Psychological disorders & Obsessive-Compulsive	Infertility & unsuccessful treatment induced stress creates disorders in hormones level & neurotransmitters, making the person prone to psychological disorder. High depression in women & high anxiety in men have been reported.	In the infertile couple, paying attention to adjustment mechanisms and their modification, focusing on depression & anxiety symptoms, particularly suicide & various medicinal treatments and psychotherapy.
Life quality	8	The person’s mental image about resigning to life conditions	In fact, the study items in life quality & marriage satisfaction overlap, thus to promote each of them results in the other one’s promotion.	Increasing the patients’ knowledge about the existing treatment methods, not doing invasive methods as much as possible, analyzing sexual disorders and removing them, if existing.
Psychological, socioeconomic & family supports	11	Support by consulting, the spouse, the couple’s family, removing the stigma in society, covering infertility treatments by insurance	Decreasing stress, accepting childless life by the couple, assurance in terms of financial affairs provision to continue treatment.	Education to families on how to behave with the infertile couple, using media to promote infertility assisting methods in society, political support of research plans in diverse infertility treatments & consultation at infertility treatment centers.
Sexual function	8	Erection disorder, ejaculation praecox, arousal & orgasm disorder, lowered sexual desire	Because of self-concept damage, self-confidence, masculinity & femininity feeling, feeling deficiency.	Examining depression, training the question goals in creating a sexual relationship.

**Table 3:** List of criteria for assessing the quality of studies on marital relationship in the in infertile couples

Studies	Criteria for methodological assessment of study quality																Score

	A	B	C	D	E	F	G	H	I	J	K	L	M	N	O	P	
Moura-Ramos M et al. (2011)	+	+	+	+	+	-	-	+	+	+	-	-	+	-	-	+	**10**
Kalkhoran LF et al. (2011)	+	+	+	+	+	+	+	-	-	+	-	-	+	-	+	+	**11**
Peterson BD et al. (2009)	+	+	+	+	+	+	+	-	+	+	+	+	+	+	-	+	**14**
Sun T-YL et al. (2000)	+	+	+	+	+	+	-	-	-	+	-	-	+	-	-	+	**9**
Seif D et al. (2001)	+	+	+	+	+	-	+	+	-	+	-	-	+	-	-	-	**9**
Shakeri J et al. (2006)	+	+	+	+	+	-	-	+	-	+	-	-	+	-	-	-	**8**
Heidari P et al. (2010)	+	+	+	+	+	+	+	+	-	+	-	+	+	-	-	-	**11**
Faria DEPd e t al (2012)	+	+	+	+	+	+	-	+	-	+	-	-	+	-	-	+	**10**
Sydsjö G et al. (2005)	+	+	+	+	+	+	-	+	+	-	-	-	+	-	+	+	**11**
Verhaak CM et al. (2007)	+	+	+	+	+	+	+	+	-	+	+	+	+	+	-	+	**14**
Verhaak CM et al. (2005)	+	+	+	+	+	+	+	-	-	+	+	+	+	+	-	+	**13**
Repokari L et al. (2007)	+	+	+	+	+	-	+	+	+	+	+	+	+	+	-	+	**14**
Kim K et al. (2007)	+	+	+	+	+	+	+	-	-	+	-	-	+	-	-	+	**10**
Smith JF et al. (2009)	+	+	+	+	+	+	+	-	-	+	-	-	+	-	-	+	**10**
Tuzer V et al. (2010)	+	+	+	+	+	+	+	+	+	+	-	+	+	-	+	+	**14**
Mahajan NN et al. (2009)	+	+	+	+	+	+	+	+	+	+	-	+	+	-	+	-	**13**
Valsangkar S. et al. (2011)	+	+	+	+	+	+	+	-	-	+	-	-	+	-	-	+	**10**
Güleç G et al. (2011)	+	+	+	+	+	+	+	-	-	+	-	-	+	-	+	+	**11**
Lee T-Y et al. (2001)	+	+	+	+	+	+	-	-	+	+	-	-	+	-	-	+	**10**
Holter H et al. (2006)	+	+	+	+	+	+	-	+	+	+	+	-	+	-	+	+	**13**
Hussein A et al. (2014)	+	+	+	+	+	+	+	+	+	+	+	+	+	+	+	+	**16**
Peyvandi S et al. (2011)	+	+	+	+	+	+	-	+	-	+	-	-	+	-	+	+	**11**

## 3. Results

The quality of the included studies was assessed using the criteria checklist (Table 1), which was derived from a systematic review study, 64 papers including 29 cross-sectional studies, 6 clinical trials, 7 narrative reviews, 2 systematic, 5 quality studies, 1 case-control study, 13 prospective longitudinal studies, and 1 report from WHO have been reviewed for writing the present review paper. The cross-sectional, cohort, case-control, and clinical trial studies have been analyzed in terms of quality. In conclusion, the scores earned in terms of the papers quality for 64 ones have been 6-16 where respectively 17 studies (26.5%) have been assessed as having high quality, 41 studies (64.5%) with average quality, and 4 studies (9%) with low quality. While the papers presented in the table got scores 8-16 out of 22 studies presented, 14 papers (64%) have had average quality and 8 papers have had (36%) poor quality.

**Figure 1:**
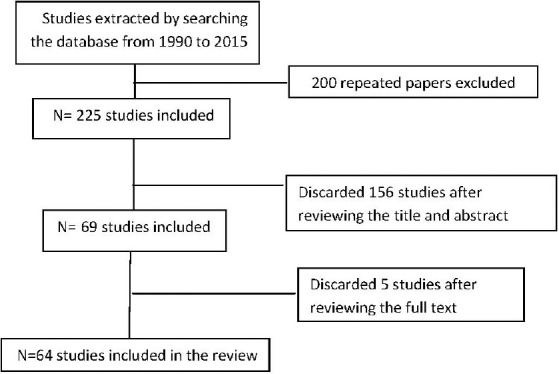
Flowchart of study selection progress

The Paper Selection Index to be included in the Figure 1.

A summary of the studies with the most important inclusion criteria to our study involving A, B, C, and E include 22 studies, and the other items are P and G with 19 studies (86%) of item P and 14 studies (64%) of item G. In the studies, estimating sample size has been different based on the research objective and study type. In the studies working on the infertile couples, sample size has been 51-379, and in the studies working on the infertile women and men separately, sample size has been 100-500 subjects. This study’s participants include the people suffering from infertility according to their physicians’ diagnosis and looking for treatment assisted fertility. The tools used to analyze the couples’ relationship in every research have been its reliability in the reported study. Generally speaking, all studies have presented at least 2 demographic variables and 2 clinical variables such as age, race, economic status, education, marriage duration, infertility type and cause, and fertility assisting treatment type.

The criteria checklist was based on an established criteria for systematic review reported in the literature (Tao et al., 2011).

Reviewing the studies performed on marital satisfaction, related findings have been classified in 6 classes: demographic factors, using fertility assisting methods, psychological health, life quality, economic, social, family support, and sexual function.

### 3.1 Demographic Factors

These factors encompass age, gender, job, education, marriage duration, infertility duration, infertility type, the previous child, family type (nuclear, extended), and income potential, that in various studies, their diverse effects have been reported like job, especially the husband’s job (due to having social status) that can accompany positive relationship with woman’s satisfaction in life. Moreover, the couples’ getting older not only does not result in their matching, but also it gradually violates their life contentment as one of the psychological compatibility elements, while another research study indicated that those cohabiting for over 9 years have reported maturity and strength more in their relationship (Faria et al., 2012; Masoumi, Poorolajal, Keramat, & Moosavi, 2013; Seif, Alborzi, & Alborzi, 2001). Education has been associated with deprivation and disappointment so that the people with higher training have less relaxation (Faria et al., 2012). Another study rejects the couple’s fertility or infertility as a factor influencing marital satisfaction and states that it is psychological and demographic factors affecting satisfaction (Kim et al., 2007). The impact of gender has been reported effective consistent with both infertility and contentment of the couple and infertility induced anxiety and depression so that in some studies, because of diverse reasons, dissatisfaction has been attributed to men (Sun, 2000; C. Verhaak, Smeenk, Van Minnen, Kremer, & Kraaimaat, 2005). In a systematic review of qualitative study on marital relationship, it has been assumed that the infertile women have unstable life compared with the fertile ones (Tao et al., 2011). About the infertility type and cause, some studies have indicated that if infertility is related to male factor, it can exert more negative impacts on the couple’s sexual affairs, and they will experience lower quality in their personal life compared with the time the infertility factor is unknown or female related. However, in this survey, male factor has not had effect on marital satisfaction (J. F. Smith et al., 2009). On the other hand, it has been stated that if the infertility factor is the male one, emotional responses get more negative since, the infertile men, emotionally speaking, suffer from more stress in their marital relationship. Then in case the male factor is considered, it is desirable to analyze sexual stresses (Tao et al., 2011; Tuzer et al., 2010). But in one study, it has been observed that infertility whether with female/male factor or both factors can lead to marriage and sexual dissatisfaction in women relative to men, while if infertility is unknown, there is no difference in women’s and men’s sexual and marital satisfaction (Lee, Sun, & Chao, 2001). In addition, infertility duration in itself can affect the couple’s psychological health; thus, it can exert effect on marital satisfaction because the research cases reported that the women with longer infertility duration come up with more depression and anxiety symptoms so that it has been asserted that depression peak symptoms pop up after 3 years of infertility diagnosis while after 6 years, the couple gets along with this issue and their depression and anxiety symptoms are slightly mitigated (Cwikel et al., 2004; Masoumi et al., 2013; Ramezanzadeh et al., 2004).

### 3.2 Assisted Reproductive Techniques

In addition to infertility itself, the medicinal based treatment induced stress. Various fertility assisting methods and their due complications can result in psychological disorders such as depression, anxiety, Obsessive-Compulsive Disorder, especially if the number of the treatment actions and negative treatment experiences are more. Then the couple’s satisfaction declines (Reis et al., 2013; Tao et al., 2011). In some people, after diagnosis or during the treatment, it is difficult to tolerate the psychological burden of infertility diagnosis or treatment so that half of the women participating in an Assisted Reproductive Technology

(ART) study mentioned it as the most stressful experience in life and displayed 4 times more depression symptoms than the control group women (Cwikel et al., 2004). On the other hand, it has been claimed that adjustment with In Vitro Fertilization (IVF) depends on diverse factors like the performing steps, and success or failure in treatment (C. M. Verhaak et al., 2007). In the women with unsuccessful treatment, less marital satisfaction has been expressed compared with those having successful treatment, that is, having a child (Monga, Alexandrescu, Katz, Stein, & Ganiats, 2004). Of course, after finishing the treatment and successive failures, the couples try to adjust with infertility (Sydsjö, Ekholm, Wadsby, Kjellberg, & Sydsjö, 2005). However, six months after the first failure, the treatment is more stressful and their depression and anxiety increases (C. Verhaak et al., 2005). The couple’s reaction to doing infertility assisting methods is seen differently. Women exhibit stronger emotional reactions than men so that higher depression is reported in them (Holter, Anderheim, Bergh, & Möller, 2006). On the other hand, a study indicated that infertility treatment success or failure cannot influence marital satisfaction as seen in ART. Even success could not be considered as the foundation for marital relationship stability. Rather, it is the infertility induced stress division between the couple that determines marital satisfaction. It means that both wife and husband suffer from infertility since both will be seeking treatment methods (Repokari et al., 2007). If after an unsuccessful ART treatment women focus on new goals in life and adjust with the new conditions, their depression and anxiety will drop, but in case of continuing treatment and not being compatible with the new conditions, they will get hurt psychologically (Greil, McQuillan, Lowry, & Shreffler, 2011; C. Verhaak et al., 2005).

### 3.3 Psychological Health

Psychological and mental health of the infertile couple affects their capability to adjust with infertility, treatment steps, pregnancy and playing the role of parents after a fruitful treatment; Adaptation strategies, personal traits, family and social supports bring about the couple’s confidence and reduce the infertility resulting from psychological distresses and can create more satisfaction in life (El Kissi et al., 2013; Hussein, 2014; Latifnejad Roudsari, Allan, & Smith, 2013; Roudsari, Allan, & Smith, 2007). Also, in another research conducted on Iranian infertile couples, it has been spotted that almost 10% of them experienced high degrees of depression and anxiety, particularly the housewives; moreover, depression prevalence in the infertile women has been reported between 5-50 %, where it has been observed that infertility duration is related to education and employment, and, finally, depression and disappointment lead to reduced marriage contentment in women (Al-Homaidan, 2011; Seif et al., 2001; Yassini, Khalili, & Hashemian, 2005). On the other hand, the study by Kalkhoran et al. has reported the infertile women’s depression and anxiety higher than that of the fertile ones. But about the marital satisfaction of these two groups, no meaningful difference has been observed (Kalkhoran, Bahrami, Farrokhi, Zeraati, & Tarahomi, 2011). The intrapersonal differences including the couple’s attachment, personality, interpersonal relationships, and social support influence the individuals’ psychological compatibility with infertility and marital adjustment (Mahajan et al., 2009; C. Verhaak et al., 2005). On the one hand, the more the couple agree in accepting tough life conditions and their perception of infertility, the higher their marital satisfaction will be (Tao et al., 2011). To accept childless life style results in marital adjustment, in particular in men (Harvey, 2008; Lee et al., 2001). Overall, more of them use problem-based coping strategies while psychologically disordered individuals apply emotion-based coping strategies more, where the former group’s individuals feel more satisfied (Peterson et al., 2009; Shakeri, Hossieni, Golshani, Sadeghi, & Fizollahy, 2006). Thus, it is recommended that the counselors analyze the couple’s adjustment mechanisms while interviewing the infertile couples and, if possible, make efforts to help them modify it (Cizmeli, Lobel, Franasiak, & Pastore, 2013).

### 3.4 Quality of Life

Factors such as age (due to better physical health), previous surgeries on genitals system, prior IVF (due to trauma), education, and bad sexual life can influence life quality (JR Chachamovich, Chachamovich, Zachia, Knauth, & Passos, 2007; Faria et al., 2012). Besides, it seems that infertility exerts equal effects on women’s and men’s life quality (J Chachamovich et al., 2009). For keeping on a comprehensive treatment for infertility, it is essential for the couple to have fulfilling life quality because by affecting sexual and marital satisfaction, it can influence the couple’s life quality (Monga et al., 2004; Valsangkar, Bodhare, Bele, & Sai, 2011). Low life quality and, finally, lack of marital satisfaction can lead to divorce (Amiri et al., 2012; Masoumi et al., 2013). Therefore, it can ultimately be concluded that life quality and marital satisfaction and sexual satisfaction can have effect on each other (Masoumi et al., 2013; Shindel, Nelson, Naughton, Ohebshalom, & Mulhall, 2008; Valsangkar et al., 2011).

### 3.5 Psychological, Socioeconomic and Family Support

Among the infertile individuals, those under family pressure have reported more severe depression than those free from this pressure; on the other hand, these individuals may face aggression by their partner or family and resort to various partners for gestation and because of unprotected sex, they may be exposed to venereal disease (Al-Homaidan, 2011; WHO, 2013). In some cultures, infertility is viewed as stigma whether in those selecting childless life style or the ones having medically diagnosed infertility problem. Accordingly, this issue creates higher stress for the infertile ones that can in turn have negative impact on their marital and sexual relationships. On the other hand, counseling can have positive effect on the person’s sexual and marital life, and at the end, the couple’s life quality (Harvey, 2008; Valsangkar et al., 2011). Being blamed by others and not being supported by the spouse can generate psychological pressure and satisfaction drop, but the family’s socioeconomic status can have positive effect on marital satisfaction as the result of building security for treatment expenses (Harvey, 2008; Seif et al., 2001). Also, it has been seen that psychological and social supports by training and consultation can affect the infertile couple’s psychological status and reduce their stress (Bennett et al., 2014; Hamzehgardeshi et al., 2015; Heidari & Latifnejad, 2010; Niforooshan, Ahmadi, Abedi, & Ahmadi, 2006; Peyvandi, Hosseini, Daneshpoor, Mohammadpour, & Qolami, 2011; Van den Broeck, Emery, Wischmann, & Thorn, 2010; C. Verhaak et al., 2005; Wischmann, 2010).

### 3.6 Sexual Function

A study implies that there exists a positive relationship between marriage duration and the quality of sexual experiences in the infertile couples. The infertile men stated that they have more problems compared to women regarding sexual experiences quality, but no meaningful relationship has been discovered between the two control and infertile groups in terms of sexual function. Both the infertile women and men set forth problems related to mutual agreement including less agreement to express their emotions compared to the control group (Güleç, Hassa, Yalçın, & Yenilmez, 2011). Most of the infertile couples express lack of sexual satisfaction, since infertility renders four significant effects of covering scheduled intercourse, viewing sex cones as a means to an end and not as an end in itself, and loss of privacy before the physician. The act of intercourse itself reminds the couple of their infertility (Greil, Porter, & Leitko, 1990). The experienced negative emotions in women bring about sexual disorder in sexual interest, sexual desire, sexual arousal, orgasm, sexual satisfaction, and sexual activity, while in men they more lead to delayed ejaculation and erection (Faria et al., 2012; Millheiser et al., 2010; Shindel et al., 2008; N. K. Smith, Madeira, & Millard, 2015; Wischmann, 2010). On the other hand, good sexual satisfaction indicates physical and psychological health resulting from marriage contentment (Harvey, 2008; Tao et al., 2011). Improved sexual satisfaction and marital adjustment in women with higher body image have proved to be one of the solutions to reduce sexual problem and marital conflict in infertile women (Karamidehkordi & Latifnejad Roudsari, 2015).

## 4. Discussion

The present study focuses on factors associated with marital satisfaction in the infertile couples. This review research aims to make the treatment team aware of what factors influence marriage satisfaction, since marital satisfaction can highly help treatment goals’ development. In our extracted studies, we found out a great number of differences affecting various factors in marital satisfaction. Our findings imply that the demographic factors can be of the important marital satisfaction influencing factors, like job that can exert positive effect on the couple’s contentment because of social position; also, infertility itself affects satisfaction by creating stress, the fear from treatment complications, and stigma, while study (Kim et al., 2007) rejects the direct impact of infertility on satisfaction and contends that it is due to the psychological and demographic factors that infertility brings about marital satisfaction. We believe that male factor infertility has come up with further negative responses in sexual relationships than the female one and finally, affects marital satisfaction, because we discovered that compared to women, men are less inclined to express their infertility problems and, as a result, seek treatment less and tolerate more psychological burden. On the other hand, men consider infertility almost equal to reduced masculinity and fear it in society as stigma. The studies (J. F. Smith et al., 2009; Tao et al., 2011; Tuzer et al., 2010) also verify this point.

In addition, we found out that infertility duration (ranging from 3 to 6 years with the highest destruction effect), the type and the number of the failures in treatment can influence satisfaction so that the more the failure times and the more invasive the treatment, the higher the destructive impacts on satisfaction (Cwikel et al., 2004; Ramezanzadeh et al., 2004; C. M. Verhaak et al., 2007). While the study suggested that even successful infertility treatment cannot result in marital satisfaction in the infertile couples (Repokari et al., 2007), we have drawn this conclusion that the more stable the person in terms of personality trait and the healthier psychologically and spiritually the better they would perform in acceptance and adjustment, which is corroborated by the findings of this study (Lee et al., 2001; Mahajan et al., 2009; Roudsari & Allan, 2011; Shakeri et al., 2006).

We believe that quality of living and marital satisfaction can bilaterally impact on one another. The ones with higher quality of living are also availed of more marital satisfaction. Likewise, the ones with higher marital satisfaction overestimate their quality of living. Studies are in accordance with our statements (J Chachamovich et al., 2009; Valsangkar et al., 2011).

Another important factor we found in a review of studies is the importance of different kinds of supports on marriage life in infertile couples so that infertile couples who are provided with psychological, social, economic, and family support show more satisfaction than the infertile couples who are not under any support. In this case we agree with most of the articles were accepted.

The studies (Masoumi et al., 2013; Valsangkar et al., 2011) are consistent with our findings.

We found that in addition to sexual function, the quality of sexual relationships can affect the satisfaction of couples, because most of the time, infertile couples do not have problems in sexual function, but due to mental stress and conflict on childbearing their sexual relationship is impaired which contributes to marital dissatisfaction. However, some studies have reported that infertility can induce such sexual dysfunctions as decreased libido, arousal, and orgasm dysfunction in women and premature ejaculation and erectile dysfunction in men. These cases will bring a lot of problems in marital satisfaction (Harvey, 2008; Seif et al., 2001; Valsangkar et al., 2011). The present review showed that sexual relationships’ quality can play role as a factor influencing marital satisfaction, not only sexual function. Because most of the time the couple have problems in sexual function rather due to stress, their sexual relationships quality gets impaired leading to lack of marital satisfaction. But some studies have stated that infertility can create sexual function disorder in women in the form of lower sexual desire, disorder in arousal and orgasm and in men, as disorder in erection, and early ejaculation (Faria et al., 2012; Millheiser et al., 2010; N. K. Smith et al., 2015).

## 5. Conclusion

The results of this review article depicted that various factors play role in creating marriage life satisfaction in an infertile couple so that paying attention to them can play an important role in continuing their treatment. Thus, to identify such factors is considered essential in their treatment protocol highly based on culture. Of the drawbacks of this research is that is has tried at best to employ the studies belonging to diverse countries with different cultures. And also the number of the papers was markedly limited.

## 6. Recommendations

**Methodological Recommendations:** It appears worth mentioning that this research has been a part of master’s degree project and there has been special time constraint. Thus, for doing any research on this target community, it is recommended first to conduct a comprehensive review study to extract the related factors to focus on the most significant relevant factor.

**Psychological, relational, and sexual recommendations:** Since couples eventually accept infertility and being childless, they will reduce stress and enhance acceptance and marital satisfaction. Therefore, the research team suggests a couple turn to the assessment and treatment of marital dissatisfaction in Infertile Couple. Sexuality is paradoxical-sexual dysfunction, especially lowered desire, and it increases as infertility problems continue. In addition, males feel more shameful, but deny their infertility and do not seek psychological support. Thus, comprehensive psycho-bio-social approach in couple therapy and counseling can improve sexual, marital satisfaction and quality of life in infertile couple.

**Implications for Practice**

Performing this study helps the health providers and the physicians, and psychiatrists dealing with such a group of people to know that addressing the couple’s problems is really significant and can remarkably influence their treatment process.

**Implications for Research**

Performing such studies in every community seems necessary before running the intervention since reviewing the prior studies can pave the ground to analyze the factors related to the subject in various communities and to design the effective interventions consistent with our community.
